# First-Trimester Maternal Serum Levels of sFLT1, PGF and ADMA Predict Preeclampsia

**DOI:** 10.1371/journal.pone.0124684

**Published:** 2015-04-23

**Authors:** Zheng Bian, Chenzi Shixia, Tao Duan

**Affiliations:** Department of Obstetrics and Gynecology, Shanghai First Maternity and Infant Health Hospital of Tongji University, Shanghai, China

## Abstract

**Background:**

Placental growth factor (PGF), soluble fms-like tyrosine kinase 1 (sFLT1) and asymmetric dimethylarginine (ADMA) are involved in the pathogenesis of preeclampsia. Abnormal maternal sFLT1, PGF and ADMA levels are detectable weeks before the onset of preeclampsia.

**Objective:**

To investigate sFLT1, PGF and ADMA in the first trimester of pregnancy as predictors of preeclampsia.

**Methods:**

In this prospective nested case-control study, 740 pregnant women enrolled at 12–16 weeks of gestation and followed up until 6 weeks after delivery at the Shanghai First Maternity and Infant Health Hospital of Tongji University between January 2010 and December 2012. Forty-four women developed preeclampsia. Urinary proteins were measured using 24-hour collection or dipsticks. sFLT1, PGF and ADMA were measured by ELISA in the first trimester. Pulsatility index (PI) was measured by Doppler ultrasound in the second trimester.

**Results:**

First-trimester serum sFLT1 and ADMA levels of women who developed preeclampsia were significantly higher compared with women with normal pregnancies (sFLT1: 0.321±0.023 vs. 0.308±0.019 ng/ml, P = 0.001; ADMA: 0.86±0.16 vs. 0.68±0.20 μM, P<0.001). First-trimester serum PGF levels of women who developed preeclampsia were significantly lower than in women with normal pregnancies (115.72±32.55 vs. 217.30±74.48 pg/ml, P<0.001). Multiple logistic regression and receiver-operating characteristic curves identified first-trimester PGF and ADMA to be sensitive and selective predictors of preeclampsia (area under the curve [AUC]: 0.902), as well as second-trimester uterine artery pulse index (AUC: 0.836).

**Conclusion:**

In the first trimester, maternal serum sFLT1, PGF and ADMA levels, as well as second-trimester uterine artery PI, could predict preeclampsia.

## Introduction

Preeclampsia is a common form of hypertension experienced during pregnancy and complicates 5 to 8% of all pregnancies [1–3]. Risk factors for preeclampsia are preexisting hypertension, nulliparity, multiple gestation, >20 weeks of gestation and being near term [1, 4]. Preeclampsia may be harmful to both the women and her child, and may result in maternal mortality (15–20% in developed countries, 40–80% in developing countries), acute and long-term morbidities, perinatal deaths, preterm birth, and intrauterine growth restriction [1, 4–6]. Clinically, preeclampsia is characterized by hypertension, proteinuria and edema after 20 weeks of gestation and can be complicated by renal failure, pulmonary edema and coagulopathy. Preeclampsia can also progress to the HELLP (hemolysis, elevated liver function and low platelet) syndrome and seizures (eclampsia) [7].

Although the etiology of preeclampsia is still unclear, recent studies suggest that placentation is a major cause. Preeclampsia occurs only in the presence of the placenta, with or without a fetus, as in the case of hydatidiform mole. Preeclampsia is a two-stage process [8, 9]. The first stage is characterized by abnormal placentation, and the second stage is characterized by hypertension and proteinuria. Individuals with a predisposition to vascular insufficiency, such as diabetes mellitus, thrombophilias, systemic lupus erythematosus, chronic hypertension and obesity are at a higher risk of preeclampsia [10]. Women with higher placental mass and comparatively less placental blood flow are also at elevated risk of preeclampsia [11].

Previous studies identified risk factors for preeclampsia, but mostly failed to obtain a comprehensive panel of biomarkers that could predict the appearance of preeclampsia with high sensitivity and specificity [12]. Nevertheless, high triglyceride levels [13], high soluble fms-like tyrosine kinase 1 (sFLT1) and low placental growth factor (PGF) levels [14, 15], high hynurenic acid levels [16], low adiponectin levels [17], vascular biomarkers such as asymmetric dimethylarginine (ADMA), nitric oxide and vascular endothelial growth factor [18], and high HbA1c levels [19] were suggested as potential biomarkers for predicting the risk of developing preeclampsia.

Despite the fact that there is no actual cure for preeclampsia, identifying women at higher risk of developing preeclampsia is important in order to treat these women in a timely fashion and to minimize complications to them and their child. The aim of the present study was to identify biomarkers that could be predictive of preeclampsia. Based on previous studies, we selected serum levels of PGF, sFLT1 and ADMA.

## Subjects and Methods

### Participants

This prospective nested case-control study was carried out between January 2010 and December 2012. We screened 770 women who received prenatal care in Shanghai First Maternity and Infant Health Hospital of Tongji University. Women were enrolled during a routine visit between 12+0 and 16+0 weeks of gestation. All participants and their partners were Chinese. Exclusion criteria were histories of 1) hypertension; 2) liver or renal diseases; 3) diabetes mellitus; 4) cardiovascular or vascular diseases; 5) thrombophilia; or 6) systemic lupus erythematosus. Ethics committee approval was obtained from the Shanghai First Maternity and Infant Hospital of Tongji University, and women provided written informed consent.

### Data collection

Information was collected regarding the current pregnancy including age, disease and obstetric history, drug treatments, height, weight, blood pressure and proteinuria. Delivery age and birth weight were also collected by the same study team of physicians and nurses.

### Blood samples

The occurrence of preeclampsia is related to the changes in the structure of the placenta. Normal placentation involves the invading course of trophocytes to the matrix of the spiral artery, and this process completed between the 14–16^th^ to the 18–20^th^ weeks of gestation [20]. Therefore, we selected the timing of blood sample collection at the 12–16^th^ week, which is the first routine prenatal examination in China. Blood samples were centrifuged at 3,000 rpm at 4°C to obtain the serum. Samples were stored at -80°C until analysis.

### Measurement of serum levels sFLT1, PGF and ADMA

Serum sFLT1, 8-iso-prostaglandin F 2α (PGF2α) and ADMA were measured using solid-phase sandwich enzyme-linked immunosorbent assay (ELISA) kits (R&D Systems, Minneapolis, MN, USA) by technicians who were blinded to the clinical outcome. All samples were run in duplicate. If there was a variation of more than 25% between duplicates, the assay was repeated a third time, and the average was reported. Inter-assay variability coefficients for PGF, sFLT1 and ADMA ELISA assays were 13.2%, 12.6% and 10.6%, respectively.

### Pulsatility index evaluation by Doppler ultrasound

Since the whole recasting of the placenta structure is completed at about 24 weeks of gestation [21], we performed PI measurement at 20–24 weeks. Doppler ultrasound of the uterine arteries was performed in the second trimester of pregnancy (20–24 weeks after conception) by experienced sonographers. Uterine artery PI was calculated as the mean PI from three similar consecutive waveforms. All examinations were carried out transabdominally using a C3-5 MHz curvilinear transducer (Envisor 2540A, Philips Medical Systems, China).

### Follow-up and Preeclampsia definition

All women were followed up for 6 weeks after delivery. Preeclampsia was defined as systolic blood pressure ≥140 mmHg and/or diastolic blood pressure ≥90 mmHg on at least two occasions separated by at least 4 h after 20 weeks of gestation in previously normotensive women, with 24-hour proteinuria ≥300 mg, or two readings of at least 2+ on dipstick analysis of midstream or catheter urine specimens if 24-h urine collection was not available [5]. Mild preeclampsia was defined as a blood pressure ≥140/90 mm Hg occurring at ≥20 weeks of gestation and associated with the new onset of ≥300 mg protein in a 24 hour maternal urine collection or 1+ protein on a dipstick. Severe preeclampsia was defined as one of the following: blood pressures ≥160 mm Hg systolic or ≥110 mm Hg diastolic assessed at least twice over a six hour window; new onset of proteinuria of ≥5000 mg in a 24 hour period; oliguria <500 mL in 24 hours; cerebral or visual disturbances, pulmonary edema or cyanosis; epigastric/upper quadrant pain; impaired liver function, thrombocytopenia, or intrauterine growth restriction. Severe preeclampsia that occurred before 34 weeks of gestation was defined as early-onset severe preeclampsia.

For the control group, we randomly selected women who had absolutely no pregnancy and obstetric complications (such as no change in blood pressure and urinary proteins), and who had a similar gestational age at the time of blood sampling.

### Statistical analysis

Data are expressed as means ± SD. The Mann-Whitney unpaired test was used for comparison of non-normally distributed data or data with heterogeneity of variance. Normally distributed data with homogeneity of variance were compared using independent samples t-tests. Multiple logistic regression analysis was used to identify combined diagnostic serum markers. The sensitivity and specificity of different cut-offs for each variable in detecting preeclampsia were calculated and depicted as receiver-operating characteristics (ROC) curves. SPSS 17.0 (SPSS Inc, Chicago, IL, USA) was used for data analysis. MedCalc (version 10.4.7.0; MedCalc, Mariakerke, Belgium) was used to perform the ROC. All statistical tests were two tailed with a significance level set at 5%.

## Results

### Patients' characteristics

**Fig 1:**
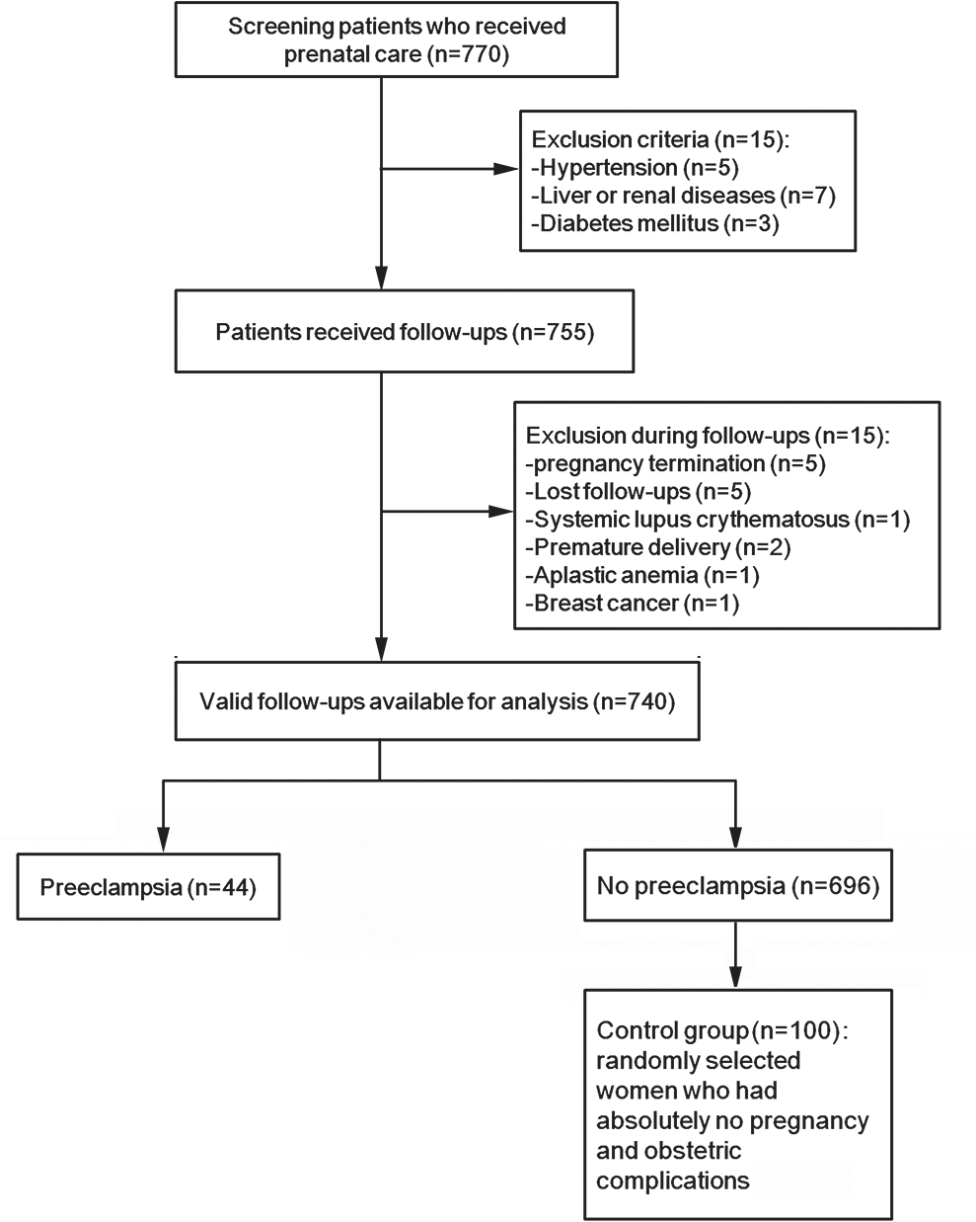
Study flowchart.

We screened all women (n = 770) who received prenatal care in Shanghai First Maternity and Infant Health Hospital from January 2010 to December 2012. According to the exclusion criteria, 15 women were excluded because of hypertension (n = 5), liver and renal diseases (n = 7) and diabetes (n = 3). Therefore, 755 women were followed up. Five women were lost to follow-up. Five women had their pregnancy terminated because of natural or medical reasons. We also excluded 5 women who suffered from serious diseases before delivery and transfered to other hospital for further treatment: 2 women with premature delivery, 1 woman with systemic lupus erythematosus, 1 woman with aplastic anemia, and 1 woman with breast cancer. Therefore, 740 women were available for analysis. Among them, 44 women developed new-onset preeclampsia (5.8%): 27 had mild preeclampsia and 17 had severe preeclampsia (including four early-onset severe preeclampsia) (Fig 1). Four participants exhibited fetal growth restriction. Then, among the 696 remaining women, we selected 100 controls.

Participant's baseline characteristics and details of their delivery are displayed in Table 1. Compared with participants who did not develop preeclampsia, participants who subsequently developed preeclampsia had significantly higher body mass index (BMI) (21.74±2.10 vs. 20.57±2.88 kg/m^2^, P<0.01) in the first trimester. Participants who developed preeclampsia also had significantly earlier deliveries than participants who did not develop preeclampsia (36.05±2.15 vs. 38.33±1.57 weeks, *P*<0.01). Children of mothers who developed preeclampsia were also significantly smaller than children born to healthy mothers (2.91±0.33 vs. 3.14±0.43 kg, *P*<0.01). At the first trimester, there was no difference in blood pressure between the two groups (Table 1).

**Table 1:** Demographic characteristics and serological markers (sFLT1, PGF and ADMA) in the preeclampsia and control groups.

	Preeclampsia (n = 44)	Control (n = 100)	*P*-value
Maternal age (years)	28.61±2.81	29.26±2.36	0.071
Gestational age (days)	106.34±5.13	105.49±6.06	0.16
Primiparous	88.60%	79.00%	0.239
First trimester Maternal BMI (kg/m^2^)	21.74±2.10	20.57±2.88	0.002
First trimester Maternal systolic pressure (mmHg)	113.05±12.31	113.14±12.00	0.966
First trimester Maternal diastolic pressure (mmHg)	64.64±8.22	63.94±7.07	0.606
First trimester sFLT1 (ng/ml)	0.321±0.023	0.308±0.019	0.001
First trimester PGF (pg/ml)	115.72±32.55	217.30±74.48	<0.001
First trimester ADMA (μM)	0.86±0.16	0.68±0.20	<0.001
Second trimester uterine artery PI	1.19±0.20	0.88±0.33	<0.001
Maternal systolic pressure (mmHg)*	152.53±7.20	127.00±9.3	<0.01
Maternal diastolic pressure (mmHg)*	96.53±3.82	80.20±5.3	<0.01
Proteinuria (g/L)	2.0±1.5	0	<0.001
Delivery age (weeks)	36.05±2.15	38.33±1.57	<0.01
Birth weight (g)	2911.09 ±328.17	3136.85±430.90	<0.001

BMI: body mass index; PGF: placental growth factor; sFLT1: soluble fms-like tyrosine kinase 1; ADMA: asymmetric dimethylarginine; PI: pulsatility index;

*: blood pressure at diagnosis in Preeclampsia group and before delivery in control group

S1 Table shows that women who developed severe preeclampsia (n = 17) had higher second trimester uterine artery PI (1.28±0.23 vs. 1.14±0.15, P = 0.042), higher systolic pressure (155.8±6.9 vs. 149.4±5.6 mmHg, P = 0.002), higher diastolic pressure (98.4±5.5 vs. 94.5±4.8 mmHg, P = 0.019) and higher proteinuria (3.87±0.95 vs. 0.82±0.56 g/L, P<0.001) compared with women with mild preeclampsia.

### First trimester sFLT1, PGF and ADMA levels

Compared with participants with normal pregnancy, first trimester serum sFLT1 and ADMA levels of participants who developed preeclampsia were significantly higher (sFLT1: 0.321±0.023 vs. 0.308±0.019 ng/ml, P = 0.001; ADMA: 0.86±0.16 vs. 0.68±0.20 μM, P<0.001) (Table 1), and serum PGF levels were significantly lower (115.72±32.55 vs. 217.30±74.48 pg/ml, P<0.001) (Table 1).

Multivariate logistic regression analysis confirmed that high first trimester BMI and ADMA, and low first trimester PGF levels were significantly associated with preeclampsia (Table 2).

**Table 2:** Multivariate logistic regression of factors predicting preeclampsia.

Clinical factors	B	S.E.	OR (95%CI)	*P*-value
First trimester BMI	0.232	0.110	1.261 (1.018–1.563)	0.034
First trimester PGF (pg/ml)	-0.028	0.005	0.972 (0.962–0.983)	<0.001
First trimester ADMA (uM)	5.046	1.609	155.460 (6.640–3640)	0.002

BMI: body mass index; PGF: placental growth factor; ADMA: asymmetric dimethylarginine; OR: odds ratio; 95%CI: 95% confidence interval.

### ROC curves for PGF, ADMA, BMI and combined prediction of preeclampsia

**Fig 2:**
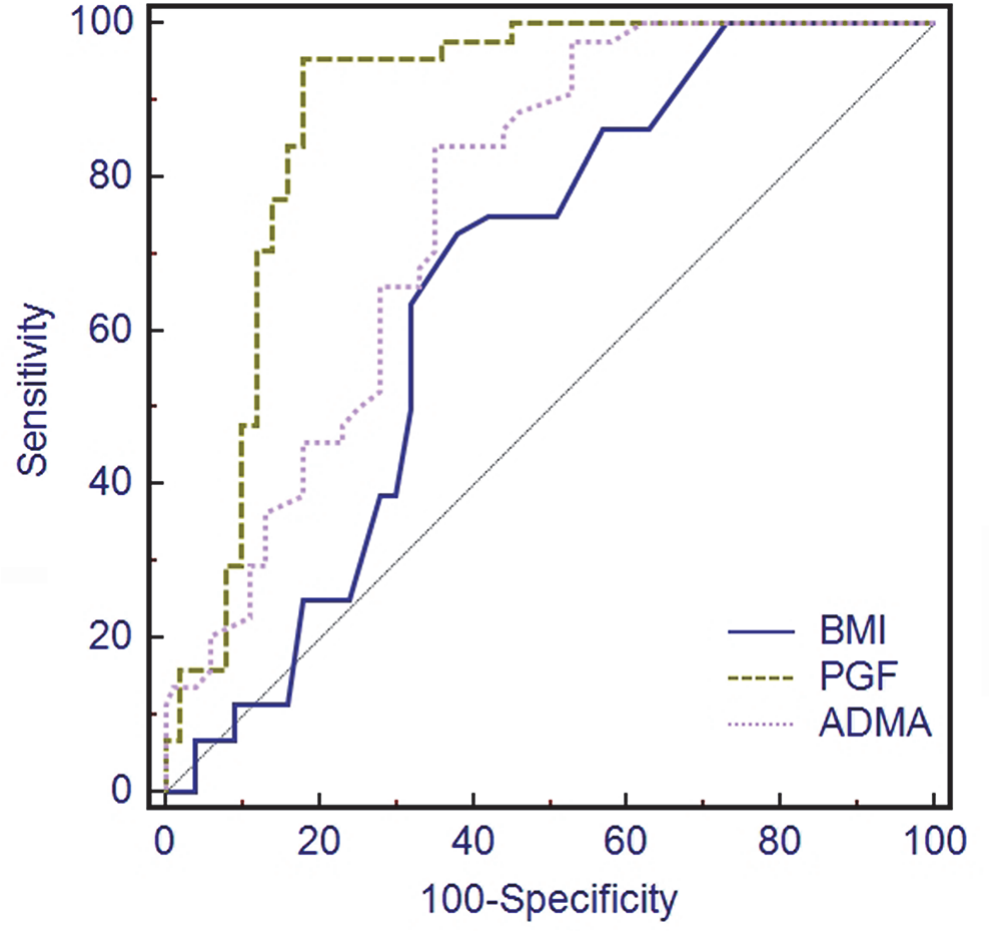
ROC curves of first trimester PGF levels, ADMA levels and BMI in the prediction of preeclampsia.

**Fig 3:**
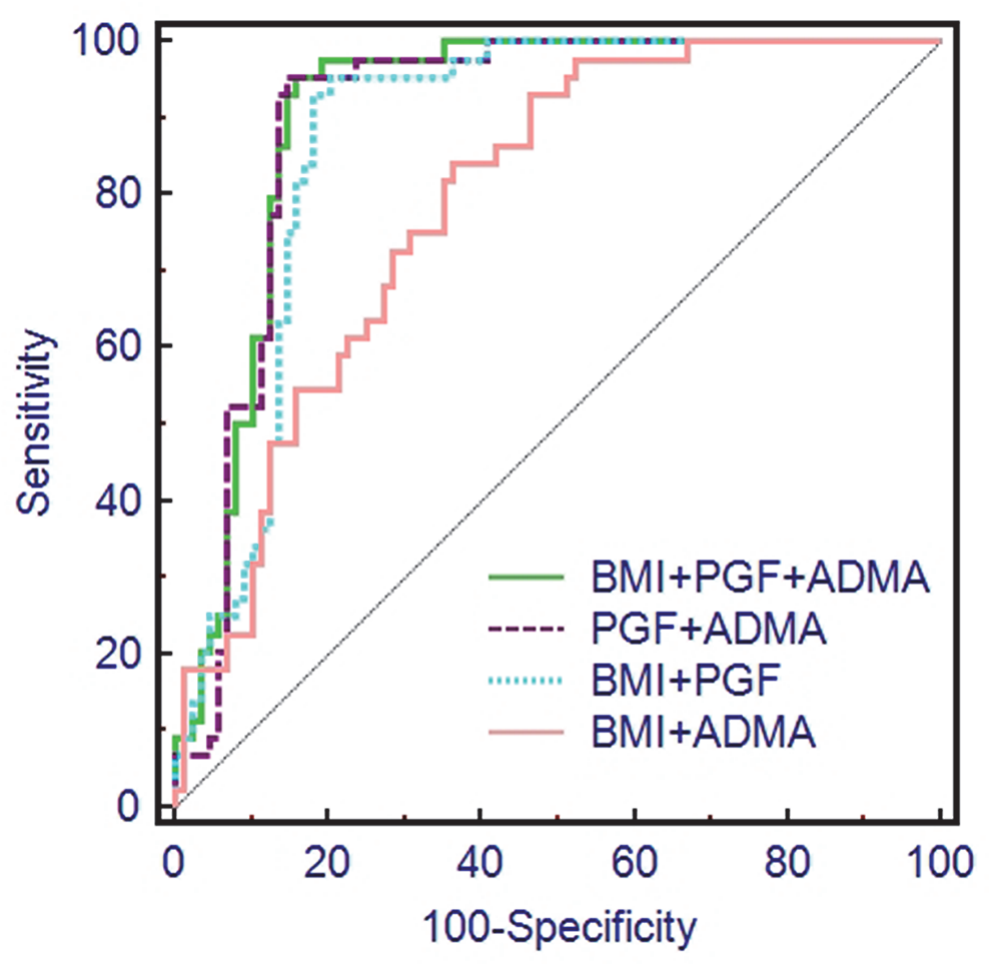
ROC curves of combinations of first trimester PGF levels, ADMA levels and BMI in prediction of preeclampsia.

Fig 2 and Fig 3 present the capacity for first trimester BMI, PGF and ADMA levels to predict preeclampsia, individually and in combination. The areas under the curves (AUC), sensitivity and specificity are presented in Table 3.

**Table 3:** Areas under the curve, sensitivity and specificity of first trimester PGF, ADMA, BMI and for prediction of preeclampsia.

Clinical factors	AUC (95%CI)	Sensitivity	Specificity	*P*-value
BMI	0.661 (0.577 to 0.737)	72.73	62.00	
PGF	0.883 (0.818 to 0.930)	95.45	82.00	
ADMA	0.763 (0.685 to 0.830)	84.09	65.00	
BMI+PGF	0.884 (0.818 to 0.932)	95.45	80.65	0.0684^a^
BMI+ADMA	0.805 (0.731 to 0.866)	93.18	56.00	0.0011^a^
PGF+ADMA	0.902 (0.837 to 0.946)	95.45	85.39	0.4697^a^
BMI+PGF+ADMA	0.909 (0.847 to 0.952)	95.45	84.62	

a: in comparison to BMI+PGF+ADMA

BMI: body mass index; PGF: placental growth factor; ADMA: asymmetric dimethylarginine; PI: pulsatility index; AUC: area under the curve; 95%CI: 95% confidence interval.

The AUC from the combination of PGF and ADMA as a predictor of preeclampsia was 0.902 (95%CI: 0.837 to 0.946), which was greater than the AUC from any single predictor or other combination of two predictors (Table 3). The AUC obtained from combining all three predictors (PGF, ADMA and BMI) was 0.909 (95%CI: 0.847 to 0.952), which was slightly higher than the AUC achieved for PGF and ADMA, but this improvement was not significant (P = 0.470, Table 3).

### Second trimester uterine artery PI as a predictor of preeclampsia

Mean second trimester uterine artery PI was significantly higher in women who later developed preeclampsia (1.193±0.20) than in women who had normal pregnancies (0.88±0.33) (P<0.001, Table 1).

**Fig 4:**
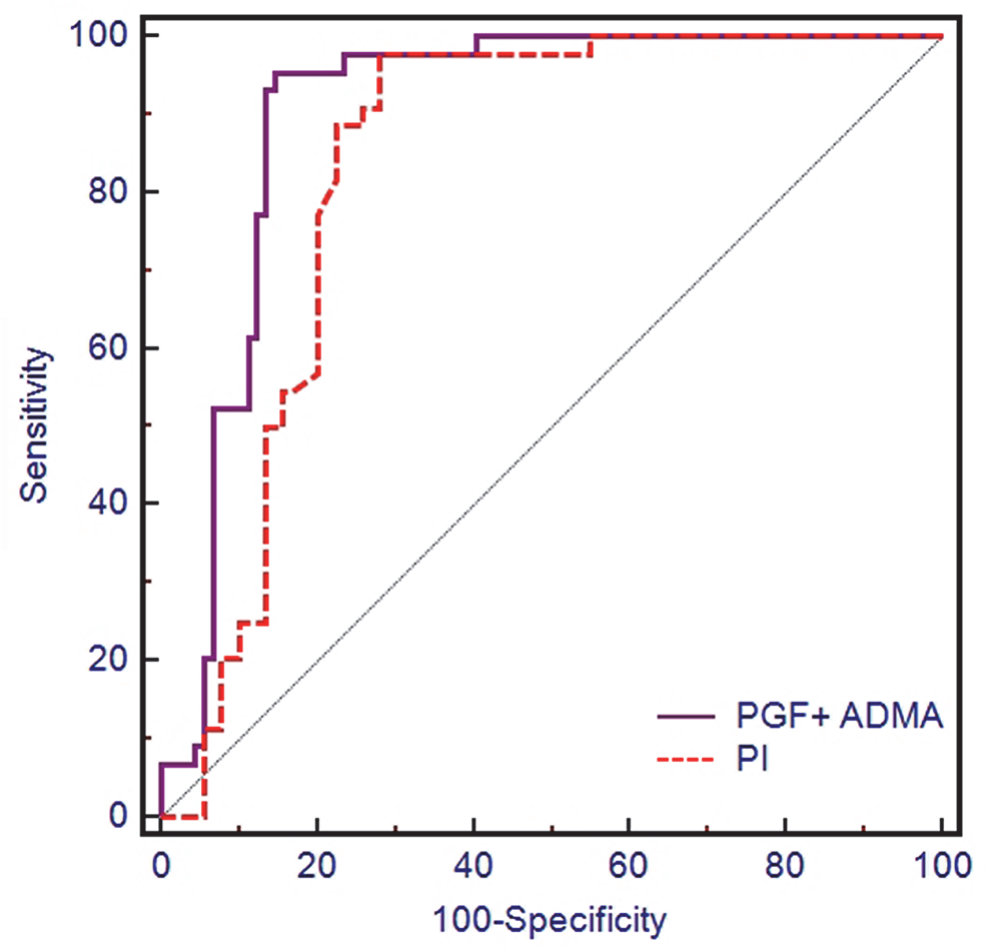
ROC curves for second trimester uterine artery PI and first trimester PGF and ADMA levels for prediction of preeclampsia.

We assessed the capacity for second trimester uterine artery PI to predict pre-eclampsia in Fig 4. The AUC obtained from the combination of PGF and ADMA as a predictor of preeclampsia, 0.902 (95%CI 0.837 to 0.946), was better than the AUC obtained from using second trimester uterine artery PI (0.836, 95%CI: 0.765 to 0.892), but this improvement was not significant (P = 0.12, Table 4, Fig 4).

**Table 4:** Areas under the curves (AUC), sensitivity and specificity of second trimester uterine artery PI for prediction of preeclampsia.

Markers	AUC (95% CI)	Sensitivity	Specificity
Uterine artery PI	0.836 (0.765 to 0.892)	97.73	73
PGF+ ADMA	0.902(0.837 to 0.946)*	95.45	85.39

*P = 0.12 compared with AUC of uterine artery PI.

PGF: placental growth factor; ADMA: asymmetric dimethylarginine; PI: pulsatility index; AUC: area under the curve; 95%CI: 95% confidence interval.

## Discussion

In the present study, 740 pregnant women were followed up from their first prenatal care visit in the first trimester until 6 weeks after delivery with the intention of identifying predictive biomarkers for preeclampsia. Forty-four women developed new-onset preeclampsia (5.8%) and were included into the case group. One hundred women with absolutely no pregnancy and obstetric complications were included in the control group.

Participants who subsequently developed preeclampsia had significantly higher serum sFLT1 levels, ADMA levels and BMI in the first trimester compared with controls. First trimester serum PGF levels of participants who developed preeclampsia were significantly lower compared with participants who had normal pregnancies. Mean second trimester uterine artery PI was significantly higher in women who developed preeclampsia. Multivariate logistic regression analysis confirmed that high first trimester BMI and ADMA and low first trimester PGF levels significantly predisposed women to preeclampsia. ROC curves identified first trimester PGF and ADMA levels in combination to be the best predictors of preeclampsia. The goal of the present study was to identify factors that could predict preeclampsia as soon as possible. Therefore, second and third trimester measurements were not included in the model.

More than 20 years ago, Doppler ultrasound was first used to measure blood flow through the uteroplacental circulation [22]. Second-trimester uterine artery Doppler has been reported to be a good predictor of severe disease. Papageorghiou *et al*. reported that increased PI (present in 5.1% of pregnancies) identified 41% of women who developed preeclampsia, and that the sensitivity for preeclampsia requiring delivery before 36, 34 and 32 weeks was 70%, 81% and 90%, respectively [23]. Consistent with the literature, the present study showed an increased uterine artery PI during the late second trimester in patients who developed preeclampsia [23–25]. However, although advances in obstetric and neonatal care have reduced the morbidity and mortality for many complications in childbirth, there have been no substantial advances in the prediction of preeclampsia for many years after the identification of PI as a predictive factor, until the discovery of some biomarkers.

Indeed, maternal serum PGF levels are significantly reduced in clinically evident cases of preeclampsia. The expected trend of PGF concentrations in normal pregnancy involves a steady increase during the first two trimesters to reach a peak at 29–32 weeks, followed by a consistent decline [26, 27]. Numerous studies have documented that in the early second trimester and as early as 10 to 11 weeks post conception, PGF concentrations in women who will develop preeclampsia are lower compared with normotensive controls. Tidwell and Su *et al*. observed that PGF in the early second trimester was a predictor of preeclampsia with sensitivity and specificity of 70–90% [28, 29].

Recent studies confirmed the observation that placental expression and serum levels of sFLT1 in preeclamptic women are increased during active disease compared with normal pregnancies [30]. A number of studies confirmed that maternal serum sFLT1 levels are correlated with gestational age [9, 31, 32]. Several studies have also measured serum sFLT1 levels between 4 and 25 weeks of gestation and confirmed that before 20 weeks, no significant difference in sFLT1 levels was observed [9], although one study did observe a trend toward a difference [32]. Only within 5 weeks of onset of hypertension and proteinuria were sFLT1 levels significantly increased [8, 30, 31]. In the present study, early trimester sFLT1 could not predict preeclampsia. This is in contradiction with Herting *et al*. who observed that the sensitivity and specificity of sFLT1 were 80% and 100% when measured in the 25–28^th^ week of gestation (late third trimester) [31].

Several studies suggested that women who develop preeclampsia are at increased risk of cardiovascular complications later in life. Maternal endothelial dysfunction is a feature of established preeclampsia, but it is unclear whether this is a cause or consequence of the disorder. Savvidou *et al*. tested the hypothesis that endothelial dysfunction and higher plasma concentrations of ADMA, an endogenous inhibitor of endothelial nitric oxide synthase, precede and contribute to the development of preeclampsia [33]. They observed that maternal endothelial function was impaired in women who eventually developed preeclampsia and that this decline occurred before the development of clinical symptoms. In the present study, ADMA levels were elevated in the first trimester in women who subsequently developed preeclampsia, indicating that this factor may provide a useful predictive marker early in pregnancy.

In the present study, BMI was an independent predictor of preeclampsia in multivariate analyses, and the ROC curve including BMI was slightly better than the one without it. However, the difference in BMI between the two groups was marginal. Indeed, a meta-analysis showed that an increased BMI before pregnancy was a likely risk factor for preeclampsia [34]. Further studies are necessary to correctly assess BMI as a predictor of preeclampsia in relation with other risk factors.

Previous studies tried to determine panels of biomarkers that could predict preeclampsia. A previous study revealed that serum levels of angiogenin, hCG, progesterone, sTNFr-2 and THF-α were higher in women who developed preeclampsia compared with women who did not, and that there was no difference in sFLT1 and PGF [35]. Furthermore, biomarkers predicting preeclampsia differed with different characteristics of the women such as diabetes, chronic hypertension, multiple gestation and a history of preeclampsia [35]. In the present study, women with hypertension or diabetes were excluded. A previous review underlined that sFLT1 and PGF were elevated in women with preeclampsia in nearly all included studies [36]. However, they did not perform analysis of combined biomarkers. Another study revealed that combining serum levels of angiogenin and PGF resulted in AUCs of 0.68–0.73 [37], which were modest compared with the present study. Finally, a study underlined that the ability of clinical characteristics alone for predicting preeclampsia in healthy women was, at best, limited, stressing the need for accurate models for predicting preeclampsia [12].

The present study is not without limitations. Indeed, it would have been interesting to examine if there were different predictive factors between early- and late-onset preeclampsia. However, only four women developed early-onset preeclampsia, preventing us to perform subgroup analyses. In addition, we only included absolutely no pregnancy and obstetric complications in the control group, which could introduce a bias. In our hospital, follow-up of pregnant women is made according to a standard method using pre-established forms for each follow-up visit. Screening for preeclampsia is standard in our hospital. All patients undergo the same screening modalities and all doctors use the same criteria for preeclampsia. In addition, since it was a prospective study, all women were followed up by the same study team of physicians and nurses. Blood pressure was not measured at the same time in the two groups. Indeed, blood pressure was measured at PE diagnosis in the PE group to avoid the influence of treatments on blood pressure. In the control group, blood pressure was measured at hospital admission for delivery, but since these women did not suffer from PE, their blood pressure should be almost unchanged during their pregnancy. Finally, sample size was small, limiting the generalization of our results, and the number of cases who developed preeclampsia vs. those who did not was very small, and we had to design a nested case control study. Further studies in larger populations are needed.

In conclusion, measurement of serum levels of sFLT1, PGF and ADMA in the first trimester could contribute to the prediction of preeclampsia. Although further larger studies are required to verify the predictive role of these serum markers, first trimester screening for serum ADMA and PGF should allow targeted application of uterine artery PI in the second trimester and allow targeted follow up of women who will subsequently develop preeclampsia.

## Supporting Information

S1 Table Demographic characteristics and serological markers (sFLT1, PGF and ADMA) in the mild and severe preeclampsia groups. (DOCX)
