# Supplementary material for: First-Trimester Maternal Serum Levels of sFLT1, PGF and ADMA Predict Preeclampsia
Source: PLoS One. 2015 Apr 23;10(4):e0124684. doi: 10.1371/journal.pone.0124684 (PMC4408038; doi:10.1371/journal.pone.0124684)
Supplement: S1 Table — (DOCX) [file pone.0124684.s001.docx]

**Table 1** Demographic characteristics and serological markers (sFLT1, PGF and ADMA) in the mild and severe preeclampsia groups.

|  | **Mild preeclampsia (n=27)** | **Severe preeclampsia (n=17)** | ***P*-value** |
| --- | --- | --- | --- |
| Maternal age (years) | 28.3±2.4 | 29.2±3.3 | 0.298 |
| Gestational age (days) | 105.8±6.7 | 106.2±4.4 | 0.820 |
| Primiparous | 88.9% | 88.2% | 1.000 |
| First trimester maternal BMI (kg/m^2^) | 21.6±2.3 | 22.0±1.7 | 0.460 |
| First trimester maternal  systolic pressure (mmHg) | 111.1±8.4 | 113.7± 9.7 | 0.346 |
| First trimester maternal  diastolic pressure (mmHg) | 62.8±7.4 | 64.5±6.5 | 0.453 |
| First trimester sFLT1 (ng/ml) | 0.32±0.02 | 0.32±0.02 | 0.991 |
| First trimester PGF (pg/ml) | 114.56± 29.14 | 117.56± 38.23 | 0.770 |
| First trimester ADMA (µM) | 0.86±0.15 | 0.88±0.17 | 0.603 |
| Second trimester uterine artery PI | 1.14±0.15 | 1.28±0.23 | 0.042 |
| Maternal systolic pressure (mmHg)* | 149.4±5.6 | 155.8±6.9 | 0.002 |
| Maternal diastolic pressure (mmHg)* | 94.5±4.8 | 98.4±5.5 | 0.019 |
| Proteinuria (g/L) | 0.82±0.56 | 3.87±0.95 | <0.001 |
| Delivery age (weeks) | 37.7±1.6 | 36.8±2.4 | 0.137 |
| Birth weight (g) | 2949±253 | 2851±430 | 0.402 |
